# Exploring factors influencing students’ self-feedback: insights from a structural equation modeling analysis using an extended theory of planned behavior framework

**DOI:** 10.3389/fpsyg.2025.1683523

**Published:** 2025-10-22

**Authors:** Yongle Yang, Zi Yan, Jinyu Zhu, Wuyuan Guo, Junsheng Wu, Bingjun Huang

**Affiliations:** ^1^Research Center for Basic Education Quality Development, School of Education, Jingchu University of Technology, Jingmen, China; ^2^Department of Curriculum and Instruction, The Education University of Hong Kong, Hong Kong, Hong Kong SAR, China; ^3^Shenzhen Baoan Haile Experimental School, Shenzhen, China

**Keywords:** self-feedback, predictors, theory of planned behavior, structural equation modeling, Chinese high schools

## Abstract

Empowering students as active agents in the feedback process is essential for students’ learning, which requires students to proactively seek, process, and use feedback to enhance their learning outcomes. Despite its critical significance in feedback research, there remains a notable gap in understanding the factors that motivate students to engage in the self-feedback process. This study applied an extended Theory of Planned Behavior (TPB) model to examine 1,311 students from mainland China regarding their self-feedback intentions and behaviors, along with crucial predictors (i.e., attitude, subjective norms, and perceived behavior control) with ten self-report scales. The psychometric properties of all scales were examined, and effects among factors were investigated using structural equation modeling. Findings reported that attitude, subjective norms, and perceived behavior control were significant predictors of students’ intentions for self-feedback, while perceived behavior control and intention notably influenced self-feedback behavior. Class climate, decomposed into individual-level (CCI) and group-level (CCG), had no significant impact on self-feedback intentions and mixed effects on self-feedback behavior. This study lays the groundwork for future efforts to promote meaningful self-feedback behavior, vital for fostering students’ metacognitive skills and lifelong learning.

## Introduction

Feedback is the process through which learners make sense of information from various sources to enhance their work or learning strategies (Boud and Molloy, 2013; Carless, 2015; Carless and Boud, 2018). Within this framework, the notion of self-feedback has been widely discussed (Berger, 1990; Hattie and Timperley, 2007) and recently framed as a practical extension of self-assessment to generate feedback for students’ educational progress (Panadero et al., 2019). It advocates a proactive agency wherein students take actions for seeking, processing, and using external information to enhance their learning outcomes (Malecka et al., 2020; Panadero and Lipnevich, 2022; Dawson et al., 2023). Such feedback engagement forms crucial components of self-feedback behavior, fostering metacognitive awareness—i.e., students’ understanding of their learning or cognitive processes (Biswas et al., 2006; Molin et al., 2020; Stanton et al., 2021). Pedagogically, effective learning necessitates students’ evaluation and subsequent use of feedback elicited from different sources (Hattie and Timperley, 2007; Boud et al., 2013; Winstone et al., 2017; Panadero and Lipnevich, 2022). Furthermore, empirical evidence supports the enabling roles of feedback on various academic facets, including performance, autonomy, commitment, engagement, and self-efficacy (Brown and Harris, 2013; Panadero et al., 2016; Carless and Boud, 2018; Yan and Carless, 2021). Henceforth, self-feedback emerges as a cornerstone of self-regulated and lifelong learning endeavors (Panadero et al., 2019; Panadero and Lipnevich, 2022). Despite its recognized significance in educational research and instructional practice, the extent to which students actively engage in the self-feedback process has not been sufficiently explored. Systematic investigations into the factors influencing students’ self-feedback behaviors remain limited.

Understanding the facilitators of students’ self-feedback behaviors is crucial for their effective engagement in the feedback process and maximizing the learning benefits of this process. This study attempts to address this research gap by investigating the factors influencing students’ intentions and behaviors regarding self-feedback, utilizing an extended framework of the Theory of Planned Behavior (TPB). This approach can elucidate the underlying mechanisms that shape self-feedback behaviors more effectively. Consequently, a clearer understanding of these mechanisms will facilitate the implementation of self-feedback as a more structured and effective instructional strategy.

### Self-feedback behaviors

The transition toward student-centered feedback frameworks has highlighted the critical importance of commitment and engagement in the self-feedback process (Malecka et al., 2020; Winstone et al., 2020; Panadero and Lipnevich, 2022). Numerous studies are attempting to delineate the self-feedback process to make it more transparent and explicit, among which two attempts are making profound contributions. A first attempt by Nicol (2020) tried to conceptualize this process as “internal feedback.” It highlighted the importance of comparing external feedback and aligning it with students’ own similar assignment experiences and learning goals. A second attempt was by Panadero et al. (2023), who have conducted several empirical studies in this space. Their series of studies has led to the self-feedback model developed from over 500 observations of self-assessment performances across subjects (Spanish, mathematics, writing) and educational levels, identifying three phases that consisted of six key processes (Panadero et al., 2024). In this model, students will first process external information through reading and recalling. They would then analyze their work by comparing it with different works. Ideally, students revise their tasks and redo them. Eventually, students could formulate reasoned judgments about their work by synthesizing insights from the earlier multiple actions. This model depicted an ideal behavioral process for performing self-assessment strategies. However, it was conducted in a “laboratory” rather than a natural classroom setting (Panadero et al., 2024, p.24). Additionally, it aimed to generate feedback information for self-assessment purposes rather than employing the processed feedback for further learning improvement strategies (Yang et al., 2025).

**Figure 1:**
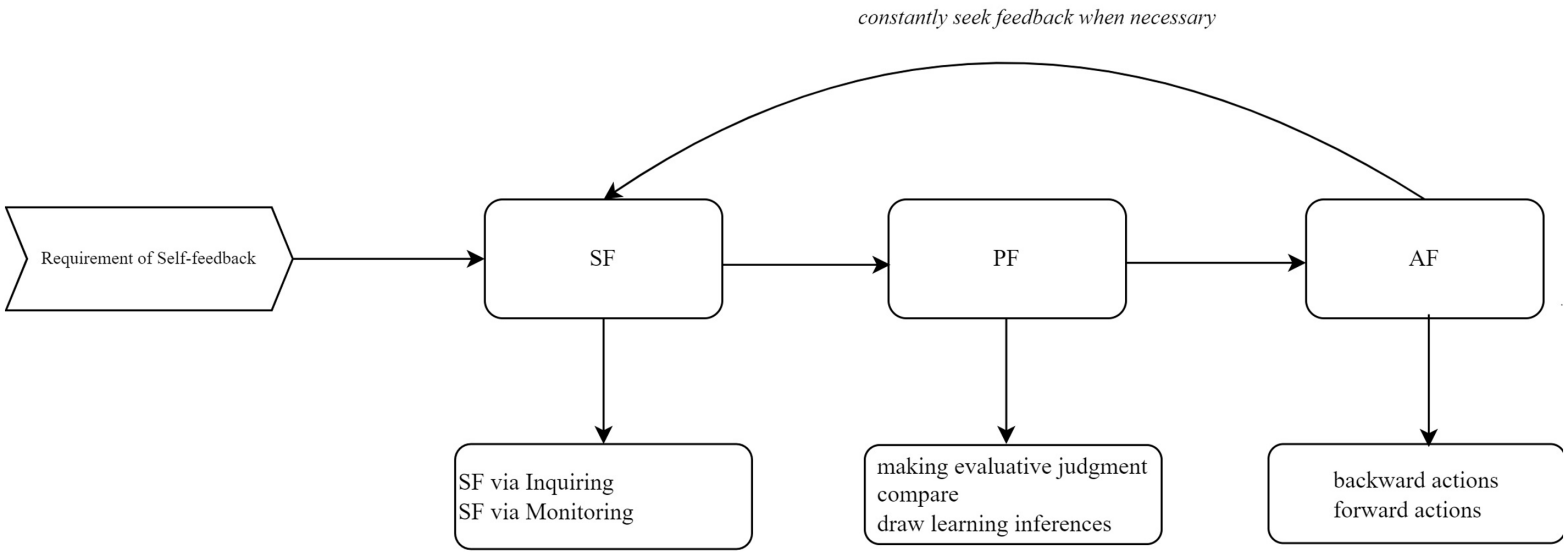
Cyclical model of self-feedback behavior. SF: seek feedback; PF: process feedback; UF: use feedback.

Notably, another recent study attempted to consider self-feedback as students’ self of agency in the feedback process; namely, students take proactive agency in seeking external information and processes and use them for their learning improvement. Self-assessment primarily generates feedback to foster deeper learning and enhance academic performance (Andrade, 2010, 2018; Yan, 2022). Therefore, building upon the behavioral framework of self-assessment established by Yan and Brown (2017), Yang et al. (2025) proposed a cyclical process model of self-feedback (Figure 1) that delineates the essential behavioral components involved. This model explicitly emphasizes students’ proactive engagement in actively seeking, processing, and utilizing feedback, thus facilitating ongoing learning and continuous performance improvement. It underscored the importance of consistently applying self-feedback strategies throughout the learning process. Ideally, this pedagogical approach views self-feedback as a continuous improvement process where students actively interact with external feedback sources, then compare and evaluate these inputs, and eventually act upon the feedback for their learning enhancement (Carless and Boud, 2018; Panadero et al., 2019; Malecka et al., 2020; Yan and Carless, 2021). Moreover, students’ proactive agency in the self-feedback process is associated with improved learning experiences, academic self-efficacy, and achievement (Panadero et al., 2017; van der Kleij, 2020; Panadero et al., 2024), though this is beyond the primary focus of the present study.

### Predictors of self-feedback behavior

Students’ self-feedback behavior requires volitional commitment and effort (Panadero et al., 2019). Henceforth, for teachers to better motivate students’ commitment and engagement in the self-feedback process, exploring the factors that could influence their behavior is critical. Numerous works of literature have been discussing how students could be better prepared and committed to the feedback process, among which the importance of managing affect, commitment to feedback as improvement, and working with emotions are emphasized (Carless and Boud, 2018; Chong, 2017; Malecka et al., 2020; Winstone et al., 2017). Specifically, Chong (2020) proposed a three-dimensional feedback literacy model comprising the contextual, engagement, and individual dimensions. It argued that contextual and personal factors would influence students’ engagement with feedback. However, the predictive effects of psychological attributes toward self-feedback behavior remain underexplored.

The Theory of Planned Behavior (TPB) (Ajzen, 1991) is considered an appropriate conceptual framework to explore the predictors within the self-feedback process, given that self-feedback is a volitional behavior. The TPB model delineates the intricate relationships among five fundamental elements associated with a phenomenon: attitudes, subjective norms, perceived behavioral control, intention, and target behavior. Specifically, people’s intention of a particular behavior is determined by three inter-connected factors: (1) attitudes, which describe the cognitive evaluation and overall assessment of target behavior; (2) subjective norms, indicating the social norms that prevail positively or negatively about the behavior; and (3) perceived behavioral control, evaluating people’s perception of their capability to execute specific behavior (Ajzen, 2020). Generally, individuals with favorable attitudes, supportive subjective norms, and confident perceived behavioral control are more likely to formulate behavioral intention. Subsequently, the target behavior will be determined by their intention and perceived behavioral control.

Numerous studies have evaluated the TPB across various academic domains, establishing its reliability and effectiveness. Meta-analyses by Godin and Kok (1996), Notani (1998), Armitage and Corner (2001), and others have consistently supported the TPB’s theoretical sufficiency. Findings indicate that attitudes, subjective norms, and perceived behavioral control are significant predictors of intention, and intention is an imperative predictor of target behavior. Recent studies across different research domains, including educational and psychological assessment, further confirm the TPB’s applicability, demonstrating its value in predicting intentions and behaviors among secondary school students in Hong Kong and mainland China (To et al., 2014; Yan et al., 2020; Wang et al., 2022; Chai et al., 2022). While TPB is widely accepted, its ability to explain behavior has often been questioned, with some researchers noting its more substantial predictive power for intention over behavior (Stanec, 2009; Yan and Cheng, 2015; Yan and Sin, 2014). Moreover, Ajzen (1991) acknowledged that there was room for additional factors to enhance the explanatory capability of an individual’s intention and behavior. In this regard, predictors such as desire (Perugini and Bagozzi, 2001), moral norms, and individual affect (Rivis et al., 2009) were suggested to be incorporated into the TPB model to improve its predictive power. Usually, the additional predictors should demonstrate their predictive effect on the target behavior, but are not included in the conventional TPB model.

The class climate was identified as a significant factor that might influence students’ intentions and self-feedback behavior (Evans et al., 2010; Marsh et al., 2012; Alonso-Tapia and Ruiz-Díaz, 2022). It describes students’ perceptions of their learning experiences in the classroom, such as how they feel in their classroom, how they interact with their teachers and peers, and how they are engaged in classroom instructional practices (Barr, 2016; Reid and Radhakrishnan, 2003). Even though each student may have different ideas and preferences about the learning environment in their classroom (Fraser, 1989; Fraser and Tregust, 1986), there is a shared learning climate within their classroom.

Students’ intentions and behavior regarding self-feedback might be significantly influenced by their perception of class climate, shaped by various stakeholders within the classroom ecosystem, including school principals, teachers, staff, and students (Trickett and Moos, 1973; Norton, 2008). The perception of class climate encompasses students’ assessment of the value placed on their contributions, the extent to which their voices are heard, and the responsiveness of peers and teachers to their inquiries and behaviors. The interconnections of these factors within the classroom environment profoundly influence students’ learning motivation (Fraser, 1989; Homana et al., 2006; Theokas and Lerner, 2006) and feedback behavior (Carless and Boud, 2018; Malecka et al., 2020; Chong, 2020). A collaborative and supportive class climate will likely foster a learning environment that facilitates students’ effective feedback strategies (Barr, 2016; Chong, 2020) and ultimately improve their academic achievements (Ellis, 2004; Frisby et al., 2014; Malecka et al., 2020; Chong, 2021). In conclusion, students’ perception of class climate appears to be a critical predictor influencing their intention and behavior of self-feedback.

**Figure 2:**
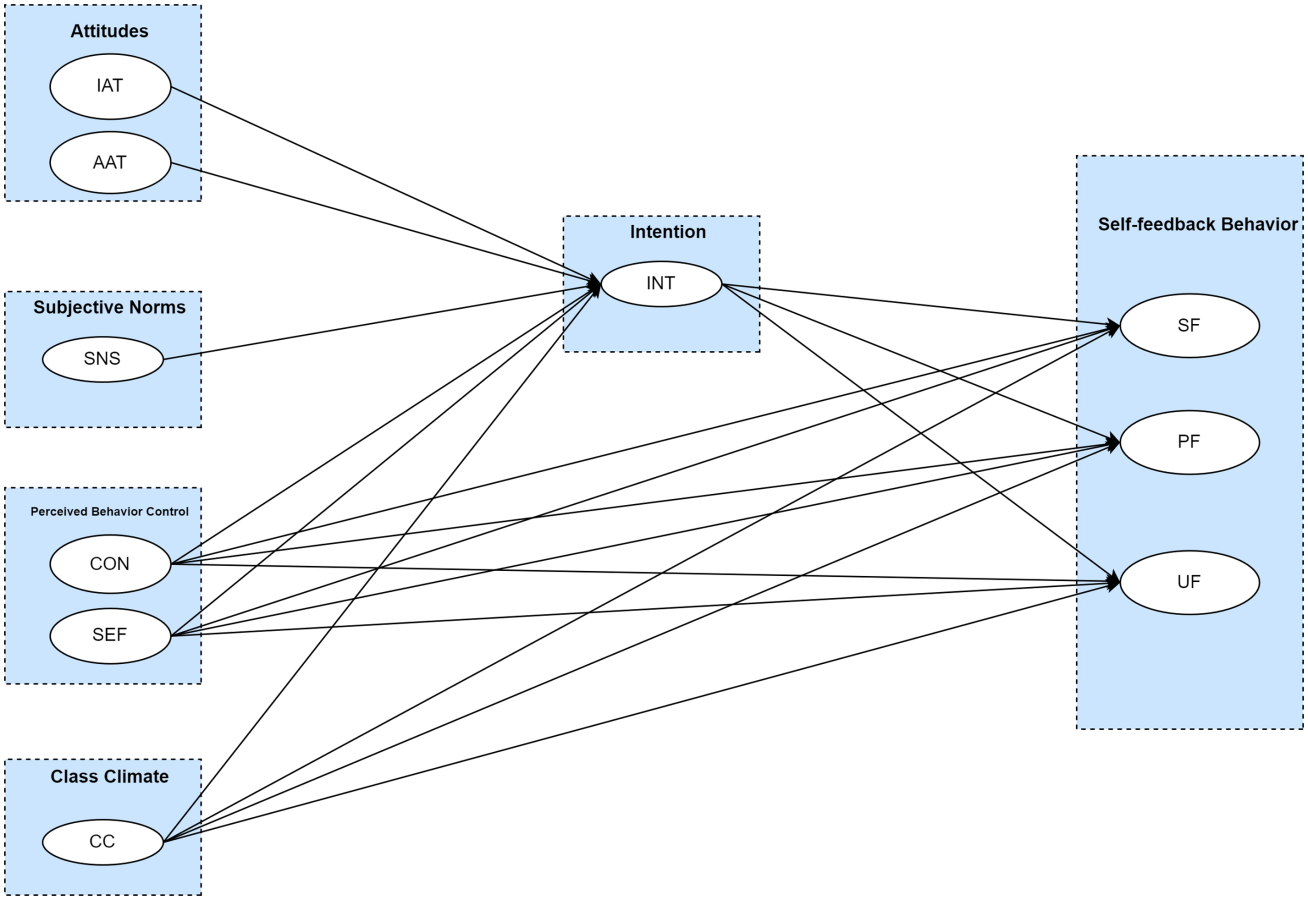
The extended model of TPB of the present study. AAT: Affective attitude; IAT: Instrumental attitude; SNS: Subjective norm; CON: Controllability; SEF: Self-efficacy; CC: Class Climate; INT: Intention; SF: Seek Feedback; PF: Process Feedback; UF: Use Feedback.

Henceforth, this study extends the TPB framework by integrating class climate as an additional component (Figure 2). Within the extended TPB framework, students’ attitudes toward self-feedback (both dimensions of affective, AAT, and instrumental, IAT) and subjective norms (SNS) were presumed as predictors of their intention (INT) to engage in self-feedback. When learners hold more favorable attitudes and perceive supportive social expectations, they are more inclined to develop stronger intentions to adopt self-feedback as a learning strategy. Furthermore, perceived behavioral control, which encompasses controllability (CON) and self-efficacy (SEF), together with class climate (CC), is expected to predict both intention and self-feedback behavior. Specifically, students who believe they have greater control over self-feedback and who experience a collaborative and supportive learning environment are expected to demonstrate higher willingness and actual practice of self-feedback strategy in their learning processes. Consequently, intention is partially mediated, influencing the relationship between student attitudes, subjective norms, and self-feedback behavior. Self-feedback behavior encompasses three interconnected actions: seeking feedback (SF), processing feedback (PF), and using feedback (UF), encompassing the entire feedback process.

### The present study: aim and research questions

The extended TPB model was employed in this study to investigate the predictive effect of predictors on students’ intentions and self-feedback behavior. The quantitative dataset was collected through a self-reported questionnaire and analyzed through structural equation modeling.

*RQ1*: What is the effect of students’ intention of self-feedback on their attitude, subjective norms, perceived behavior control, and class climate?

*H1*: Students’ self-feedback intention would be determined by attitude (H1a), subjective norms (H1b), perceived behavior control (H1c), and class climate (H1d) regarding self-feedback.

*RQ2*: What is the effect of students’ self-feedback behavior on their intentions, perceived behavior control, and class climate?

*H2*: Students’ self-feedback behavior could be predicted by their intentions (H2a), perceived behavior control (H2b), and class climate (H2c).

Another pivotal aspect addressed in this research pertained to scale development and validation, mainly focusing on the newly developed instrument, the Self-feedback Behavior Scale (SfBS; Yang et al., 2025), designed for self-feedback assessment. This study contributes further evidence using an independent dataset to examine its validity.

## Methods

### Participants

A total of 1,311 students (49% of whom were female) participated in this study, and participants were between 14 and 18 years old (See Table 1). Before the main study, the dataset was randomly divided into two sub-samples: Sample 1 (*N* = 656) was used for exploratory factor analyses (EFA) to obtain an ideal factorial structure. In contrast, Sample 2 (*N* = 655) was adopted for confirmatory factor analysis (CFA) to assess the model fit of the factorial structure. This study employed a random sampling technique to select four high schools in Shenzhen, Southern China. Two public and two private schools were randomly chosen for this study to account for differences between public and private schooling. Stratified sampling was conducted within each school across three grade levels (Grades 10–12). It is worth noting that the number of Grade 12 participants was comparatively lower than those from Grades 10 and 11, since many G12 students were under considerable academic and time pressure preparing for the Gaokao, the high-stakes national entrance examination. Finally, whole-group sampling was applied at the class level; two classes from each grade were randomly selected, and all students within those classes participated in the survey. This study intended to focus on high school students because (1) past feedback studies have been mainly implemented in higher educational settings (Malecka et al., 2020; Molloy et al., 2020), there are surprisingly few studies of feedback domains in high school, neither in mainland nor in Western settings; (2) primary and secondary school students may have difficulties accurately understanding the concepts of self-feedback behavior and thus cannot effectively respond to the questionnaire items.

**Table 1:** The demographic information of participants.

Participant	Sample 1		Sample 2		Frequency	Percentage (%)
Male	332	50.61%	337	51.45%	669	51.03%
Female	321	48.93%	315	48.09%	636	48.51%
Missing	3	0.46%	3	0.46%	6	0.46%
G10	248	37.80%	252	38.47%	500	38.14%
G11	280	42.68%	282	43.05%	562	42.87%
G12	125	19.05%	119	18.17%	244	18.61%
Missing	3	0.46%	2	0.31%	5	0.38%
Subtotal	656	100.00%	655	100.00%	1,311	100.00%

### Instruments

The TPB model requires all factors under investigation (e.g., attitude, subjective norms, etc.) to be consistent with the target behavior (Ajzen, 1991). Consequently, employing a tailored set of scales to investigate certain target behaviors is a must. The item development process in this study followed the guidelines suggested by Ajzen (2002). Self-feedback behaviors were assessed with the Self-feedback Behavior Scale (Yang et al., 2025), which was newly developed concerning available relevant instruments, such as the Self-assessment Practice Scale (Yan, 2020) and Feedback Literacy Behavior Scale (Dawson et al., 2023). Items for assessing TPB predictors were primarily adapted from previous TPB scales adopted in educational assessments, such as the Conceptions and Practices of Formative Assessment Questionnaire (Yan and Cheng, 2015; Yan et al., 2020). The translation of the instruments followed a rigorous multi-step procedure to ensure semantic and conceptual equivalence. Two professional translators first produced a Chinese version of the measures, with a third translator acting as an observer to mediate discrepancies and document the translation process. An independent bilingual expert, blinded to the original instruments, then conducted a back-translation into English to evaluate content validity. Two additional translators independently performed back-translations under the same blinded conditions to further confirm the translation accuracy. Finally, six experts, comprising two psychometricians, two educational assessment specialists, and two experienced frontline teachers, reviewed the translated items across several rounds. The panel examined each item’s clarity, cultural appropriateness, and potential bias, and eventually, the final Chinese version of the instrument was achieved. All items were administered in simplified Chinese, with students’ ratings on a 6-point Likert scale evenly spanned from 1 (Strongly Disagree) to 6 (Strongly Agree).

#### The scale of self-feedback behavior

The self-feedback behavior was assessed with the SfBS (Yang et al., 2025), which comprises 11 items describing the three sub-actions of self-feedback, including Seeking Feedback (SF) (4 items; e.g., I seek out examples of good work to improve my work.), Processing Feedback (PF) (3 items; e.g., I carefully consider comments about my work before deciding whether to use them.), Using Feedback (UF) (4 items; e.g., I can formulate my learning improvement plan after explicit inferences). These three actions were compatible with the students’ self-feedback behavioral model, which comprises seeking, processing, and using feedback (Yang et al., 2025). The validation study demonstrated that the SfBS had robust internal reliability (i.e., Cronbach’s alphas were 0.86, 0.90, and 0.85 for the three subscales) and satisfactory psychometric properties. Moreover, the heterotrait-monotrait ratio of correlations (HTMT) test, which evaluates the ratio of the inter-item correlations between factors to the inter-item correlations within a factor (Henseler et al., 2015), was 0.78, 0.79, and 0.74, respectively (Yang et al., 2025), which indicated all three sub-factors achieved adequate discriminant validity.

#### The scale of self-feedback TPB factors

Six distinct scales were developed to assess the four conventional components of the TPB model. The Affective Attitude (AAT) scale comprised four items aimed at measuring students’ emotional responses to self-feedback (e.g., “Self-feedback is interesting”). The Instrumental Attitude (IAT) scale, consisting of six items, assessed students’ evaluations of the outcomes or purposes of self-feedback (e.g., “Self-feedback helps me to understand my strengths and weaknesses”). The Subjective Norms (SNS) scale, comprising four items, delved into social norms by examining students’ perceptions of how significant others view self-feedback (e.g., “I believe my teachers want me to engage in self-feedback”). The Controllability (CON) scale, with four items, evaluated students’ beliefs regarding their control over the process of self-feedback (e.g., “I decide which method of self-feedback to utilize”). The Self-Efficacy (SEF) scale, encompassing six items, scrutinized students’ perceived capacity beliefs to implement self-feedback (e.g., “I possess sufficient knowledge to execute self-feedback”). Lastly, the Intention (INT) scale, comprising six items, explored students’ willingness or intention to engage in self-feedback (e.g., “I willingly exercise self-feedback.”).

#### The scale of class climate in self-feedback

The class climate scale (CC) (8 items) was developed to measure the degree to which students perceive a positive and supportive learning environment from both personal (e.g., I feel good and comfortable in my class) and inter-personal (e.g., in my class, everyone gets along) dimensions while practicing self-feedback. The class climate scale was contextualized into self-feedback behavior in this study, adapted from the class climate scale developed and validated by López et al. (2018).

### Data analysis

The dataset underwent preliminary screening for missing data and outliers using the R statistical computing environment (R Core Team, 2019). All participant responses were retained for subsequent analyses, as the item-level missing data for each participant remained below the recommended threshold of 5% (Tabachnick and Fidell, 2007). Normality was evaluated in accordance with guidelines provided by Kline (2015). The skewness values ranged between −0.12 and −0.95, and kurtosis values varied from −0.02 to 2.55 (Table 2). These skewness and kurtosis indices fell well within the acceptable range for future studies of structural equation modeling, which are typically set at ±3 for skewness and ±10 for kurtosis (Kline, 2015).

**Table 2:** Means, SDs, and correlations between factors.

Factors	AAT	IAT	SNS	PBC	CCI	CCG	INT	SF	PF	UF
AAT										
IAT	0.77**									
SNS	0.53**	0.61**								
PBC	0.69**	0.70**	0.60**							
CCI	0.44**	0.45**	0.49**	0.49**						
CCG	0.43**	0.48**	0.49**	0.51**	0.71**					
INT	0.69**	0.73**	0.61**	0.71**	0.50**	0.52**				
SF	0.51**	0.56**	0.47**	0.55**	0.44**	0.47**	0.62**			
PF	0.50**	0.56**	0.46**	0.57**	0.43**	0.47**	0.61**	0.78**		
UF	0.49**	0.52**	0.43**	0.53**	0.43**	0.40**	0.61**	0.71**	0.71**	
Mean	4.34	4.39	4.24	4.40	4.47	4.77	4.34	4.32	4.46	4.18
SD	0.99	0.96	0.99	0.93	0.97	0.86	1.03	0.92	0.93	1.01
Skewness	−0.48	−0.59	−0.41	−0.54	−0.55	−0.95	−0.49	−0.20	−0.36	−0.12
Kurtosis	0.74	1.00	0.62	1.03	0.66	2.55	0.58	0.17	0.49	−0.20

AAT: Affective attitude; IAT: Instrumental attitude; SNS: Subjective norm; PBC: Perceived Behavior Control; CC: Class Climate; INT: Intention; SF: Seek Feedback; PF: Process Feedback; UF: Use Feedback. *p < 0.05, **p < 0.01.

#### Exploratory factor analysis (EFA)

First, Exploratory Factor Analysis (EFA) was conducted using principal component extraction and oblique rotation (direct oblimin) on Sample 1 to explore the factorial structure of the 49-item student self-feedback behavior scale and its predictors. Several criteria were referenced during the factor extraction process, including the scree plot (Cattell, 1966; Floyd and Widaman, 1995), extracted factors with eigenvalues of 1.00 or higher (Kaiser, 1960), commonalities of each variable, and the interpretability of the extracted factors. Furthermore, items with a discrepancy of less than 0.2 between the primary and secondary factor loadings and those with factor loadings of at least 0.4 were considered cross-loading items and then removed (Schaefer et al., 2015).

#### Confirmatory factor analysis (CFA)

Second, a CFA with maximum likelihood estimation was completed on Sample 2 to confirm the EFA-suggested factor structure. Multiple criteria of model fit, such as the comparative fit index (CFI), the Tucker-Lewis index (TLI), the root-mean-square error of approximation (RMSEA), and the standardized root-mean-square residual (SRMR), were employed in the model evaluation process. Overall, models with CFI and TLI values of 0.90 or greater (Bentler, 1990), RMSEA values of 0.08 or less (Browne et al., 1993), and SRMR values of 0.05 or less (Byrne, 1998) indicated a good model fit.

#### Structural equation modeling (SEM)

Third, the entire dataset’s descriptive statistics and model fit will be examined after cross-validation of the factorial structure. Moreover, Average Variance Extracted (AVE) and internal consistency reliability were computed to examine the validity and reliability of the scales. The recommended criteria for AVE are greater than 0.50 and for Cronbach’s *α* greater than 0.70 (Fornell and Larcker, 1981; Hair et al., 2006). Additionally, the heterotrait-monotrait ratio of correlations (HTMT) analysis, which computes the ratio of the construct correlations to the correlations within a construct, was adopted to evaluate the discriminant validity; results less than 0.85 were considered acceptable (Henseler et al., 2015). Lastly, the multivariate SEM was undertaken to assess the hypothesized effect in this study. Moreover, given the possible influence of gender and grade level on students’ feedback behavior in similar studies (Irvine, 1986; Yan, 2016; Guo, 2020; Panadero et al., 2020), the moderating effects of gender and grade level on the effects between intentions and the predictors were investigated in this study. It aimed to evaluate whether the predictive effect of attitude, subjective norms, perceived behavioral control, and class climate on students’ self-feedback behavior, mediated through their intentions, varies across different genders and grade levels. All the data were analyzed using the R/lavaan package (R Core Team, 2019).

## Results

The results will be reported following the statistical analysis procedure. Initially, Exploratory factor analysis (EFA) will be employed in Sample 1, describing the formulation of the factor structure and corresponding guidelines. A confirmatory factor analysis (CFA) on Sample 2 will then assess the model fit based on the factorial structure proposed by the EFA. This aims to achieve a satisfactory factorial structure for further investigation in the main study. The main study will initially examine descriptive statistics, which include the means, standard deviations, and intercorrelations of each factor; in the meantime, reliability and validity tests will be conducted and reported. Finally, the hypothesized predictive effects regarding students’ intentions and self-feedback behaviors will be examined in response to Research Questions 1 and 2.

### EFA test on sample 1

Prior to the EFA test, the sample adequacy was examined regarding Kaiser-Meyer-Olkin (KMO) test; the result was 0.959, which was considered “extraordinary” (Field, 2018); Barlett’s test of sphericity was also reported as significant χ^2^(1458) = 27306.663, *p* < 0.001, indicating that the initial model was sufficient for EFA studies (Field, 2018). Furthermore, the initial EFA analysis implied that eight components could be extracted as their eigenvalues were more than 1.00 and contributed to 62% of the variance. At the same time, the scree plot further indicated that the slope leveled off between 9 and 10 components, suggesting that 9 or 10 components should be retained. In the meantime, the eigenvalue of the tenth component (0.972) was extremely close to 1.00. In conclusion, the EFA test suggested that 10 factors should be extracted. This result was compatible with the number of factors the extended TPB model proposed. Appendix 1 reports the rotated factor loadings of each item toward 10 factors. Moreover, the pattern matrix analysis revealed a few cross-loading items. Consequently, EFA was iterated three times, and eight items were identified as cross-loading items and eliminated. Eventually, 10 factors with 37 items were obtained (Appendix 2).

Among the 10 factors, the first factor, affective attitude (AAT) toward self-feedback, consists of four items. The second factor, the instrumental attitude (IAT) toward self-feedback, consists of three items: The subjective norm (SNS) of self-feedback comprises three items and is the third factor in the model. The fourth factor, perceived behavior control (PBC) of self-feedback, consists of six items. The fifth factor consists of three items measuring the individual perception of the class climate (CCI) of self-feedback. In comparison, the sixth factor consists of four items measuring the group perception of the class climate (CCG) of self-feedback. The seventh element consists of four items labeled as self-feedback intention (INT), and the eighth element, labeled seeking-feedback (SF), consists of four items. The ninth factor includes three items labeled processing feedback (PF), and the tenth includes three items labeled using feedback (UF). It is notable that, in the original format of the survey, Controllability (CON) and Self-efficacy (SEF) were considered two factors, while EFA revealed that they should be combined into one factor, which was named as perceived behavior control (PBC). In contrast, the class climate was initially presumed to be a single factor but suggested to be divided into two factors, CCI and CCG, respectively.

### CFA test on sample 2

CFA was undertaken to cross-validate the model’s goodness-of-fit using the 37 items from the 10-factor model resulting from the EFA to assess the factor structure further. With χ^2^(620) = 2054.706, *p* < 0.001, CFI = 0.930, TLI = 0.921, RMSEA = 0.060, and SRMR = 0.038, the CFA model fit achieved a satisfactory level.

### Descriptive result of the Main study

The means, standard deviations, and intercorrelations of each factor of the main study were reported in Table 2. Among predictors, students demonstrated the lowest agreement with Subjective Norms (SNS) (mean = 4.24) and the highest agreement with Class Climate (CC) (mean = 4.77). Likewise, for self-feedback behavior, students exhibited the lowest agreement with Use Feedback (UF) (mean = 4.18) and the highest agreement with Process Feedback (PF) (mean = 4.46). Moreover, all factors showed significant positive correlations, corroborating the previous assumption of investigating self-feedback behavior through the extended TPB model.

Moreover, to further examine the validity and reliability, the Average Variance Extracted (AVE) was computed, ranging from 0.58 to 0.77 for each factor, indicating that the convergent validity of all factors achieved an acceptable level. Meanwhile, Cronbach’s alphas for all ten sub-scales were calculated, with each factor’s Cronbach *α* value ranging from 0.80 (UF) to 0.94 (PBC), and the overall Cronbach α was 0.97, indicating that the scales achieved satisfactory internal reliability (Table 3).

**Table 3:** Item number, AVE, and Cronbach α for each resultant scale.

Scale	Number of items	AVE	Cronbach α
AAT	4	0.77	0.93
IAT	3	0.72	0.88
SNS	3	0.64	0.82
PBC	6	0.71	0.94
CCI	3	0.69	0.87
CCG	4	0.61	0.86
INT	4	0.77	0.93
SF	4	0.58	0.84
PF	3	0.74	0.89
UF	3	0.58	0.80

AAT: Affective attitude; IAT: Instrumental attitude; SNS: Subjective norm; PBC: Perceived Behavior Control; CC: Class Climate; INT: Intention; SF: Seek Feedback; PF: Process Feedback; UF: Use Feedback.

Furthermore, as reported in Table 4, the HTMT analysis for all factors achieved an acceptable level of less than 0.85, according to Henseler et al. (2015). It indicated that the factors were meaningfully differentiated, and cross-loading items did not cause significant interconnections among factors.

**Table 4:** HTMT ratio of correlation values between factors.

Factors	AAT	IAT	SNS	PBC	CCI	CCG	INT	SF	PF	UF
AAT	–									
IAT	0.83	–								
SNS	0.60	0.69	–							
PBC	0.75	0.74	0.66	–						
CCI	0.49	0.50	0.57	0.53	–					
CCG	0.49	0.53	0.56	0.56	0.82	–				
INT	0.74	0.79	0.70	0.74	0.55	0.58	–			
SF	0.58	0.62	0.55	0.60	0.50	0.54	0.69	–		
PF	0.53	0.58	0.47	0.60	0.47	0.53	0.63	0.81	–	
UF	0.57	0.60	0.53	0.60	0.52	0.48	0.69	0.83	0.77	–

AAT: Affective attitude; IAT: Instrumental attitude; SNS: Subjective norm; PBC: Perceived Behavior Control; CC: Class Climate; INT: Intention; SF: Seek Feedback; PF: Process Feedback; UF: Use Feedback.

### Structural equation modeling (SEM)

Before the SEM study, the model’s goodness-of-fit in the main study was examined using the 37 items resulting from the cross-validation study. With χ^2^(989) = 2087.099, χ^2^/*df* = 2.11, *p* < 0.001, CFI = 0.993, TLI = 0.993, RMSEA = 0.069, and SRMR = 0.060, it indicated that the measurement structure of the main study had achieved a satisfactory level.

**Figure 3:**
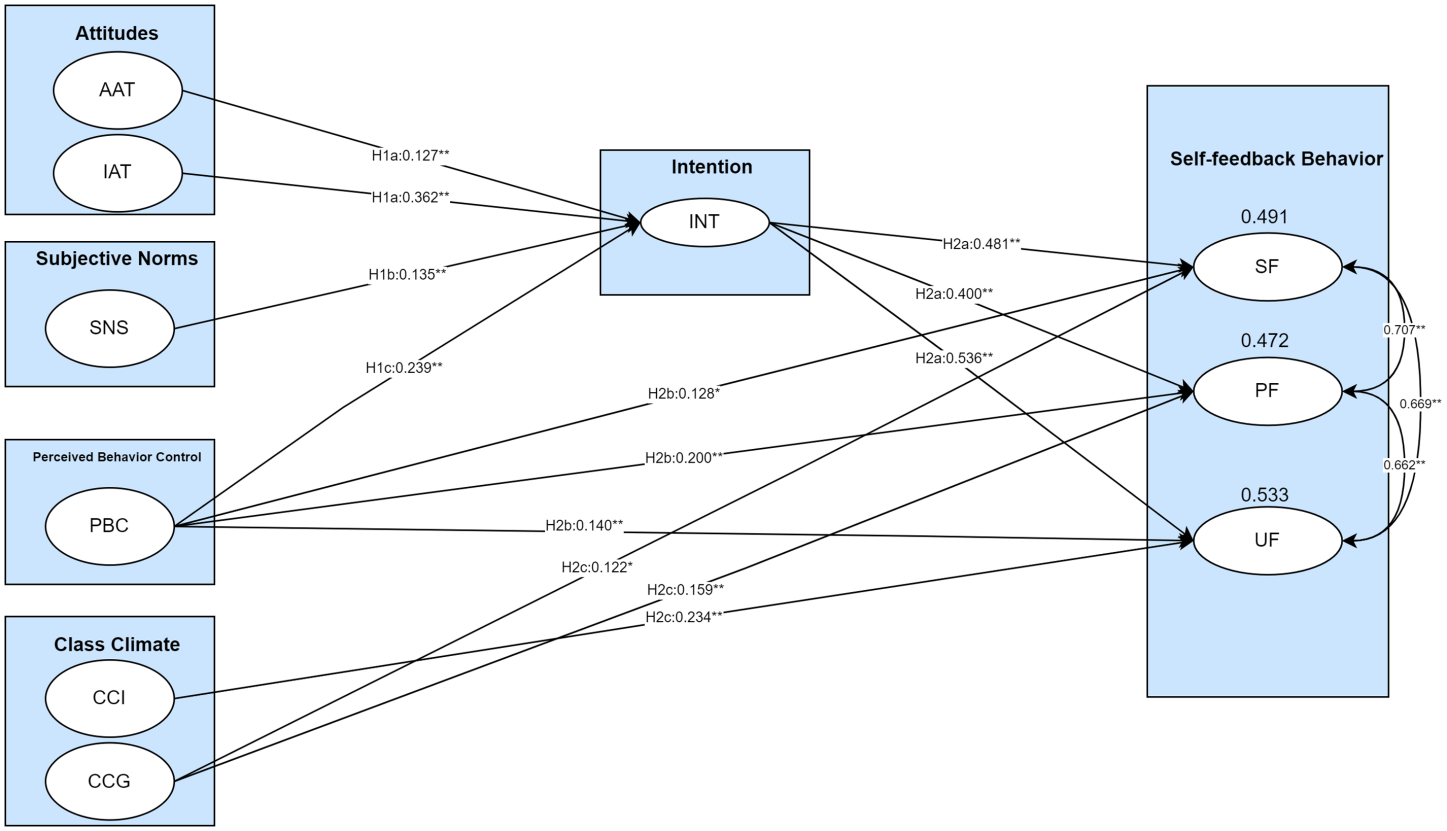
SEM analysis of the extended TPB framework. Only significant paths are indicated in the figure. AAT: Affective attitude; IAT: Instrumental attitude; SNS: Subjective norm; PBC: Perceived Behavior Control; CCI: Class Climate at Individual Level; CCG: Class Climate at Group Level; INT: Intention; SF: Seek Feedback; PF: Process Feedback; UF: Use Feedback. All predictors are correlated but not shown in this figure to avoid a messy presentation. *p < 0.05, **p < 0.01.

The effect of predictors on students’ self-feedback intention and behavior was investigated using structural equation modeling (SEM). Moreover, the possible moderating effects of students’ gender and grade level on the target effects were also examined. The standardized regression coefficients are reported in Figure 3.

### Report on the predictive effects on intention (RQ1)

Three original TPB factors (attitude, subjective norms, and perceived behavior control) significantly predicted students’ self-feedback intention. It implied that three hypotheses, H1a, H1b, and H1c, were supported. IAT had the most substantial predictive power (*β* = 0.36), whereas AAT was the least powerful predictor (*β* = 0.13). However, the factor of class climate demonstrated no significant predictive power over students’ intention of self-feedback, which indicated that hypothesis H1d was not supported. The magnitude difference between significant predictors further revealed that the predictive power of instrumental attitude (IAT) and perceived behavior control (PBC) was stronger than Affective attitude (AAT) and subjective norms (SNS). Additionally, a total of 70.5% variance in students’ intentions could be explained by all predictors.

Moreover, the potential moderating analysis of gender and grade levels revealed that neither gender nor grade level significantly moderated the effects between students’ intentions and their predictors. It was implied that the impact of attitude, subjective norms, perceived behavioral control, and class climate on students’ self-feedback behavior via their intentions remained consistent across different genders and grade levels.

### Report on the predictive effects on self-feedback behavior (RQ2)

All three self-feedback actions (Seek Feedback, Process Feedback, and Use Feedback) were robustly determined by their intention (mean *β* = 0.47) and moderately predicted by PBC (mean *β* = 0.16). This implied that hypotheses H2a and H2b were supported in this study. As for the magnitude differences between the regression coefficients, the predicting power of INT onto Use Feedback was more substantial than that of Process Feedback and Seek Feedback. Moreover, CCI had no significant predictive power on Seek Feedback and Process Feedback, while it demonstrated significant predictive power on Use Feedback. In contrast, CCG had significant predictive power over Seek Feedback and Process Feedback but no significant predictive power over Use Feedback. That is to say, the hypothesis H2c was partially supported. The proportion of explained variance of self-feedback actions varied from 47.2 to 53.3%.

### Report of indirect effects on self-feedback behavior

Table 5 reports the indirect effects of each action of self-feedback. It was found that the three conventional TPB predictors had an indirect effect on students’ self-feedback behavior mediated by intention; the indirect effect coefficients ranged from 0.05 to 0.19, and the mean coefficient was 0.10. In contrast, the predictors of class climate at both the individual and group levels (CCI and CCG) produced no significant effect on any self-feedback action.

**Table 5:** Indirect effects on self-feedback behavior.

Indirect effect	Std. Est	SE	*z*	*p*
Seek feedback pathway				
AAT → INT → SF	0.061	0.016	3.794	0.000
IAT → INT → SF	0.174	0.024	7.784	0.000
SNS → INT → SF	0.065	0.015	4.197	0.000
PBC → INT → SF	0.115	0.017	6.590	0.000
CCI → INT → SF	0.018	0.020	0.915	0.360
CCG → INT → SF	0.037	0.022	1.811	0.070
Total	0.780	0.034	23.283	0.000
Process feedback pathway				
AAT → INT → PF	0.051	0.014	3.730	0.000
IAT → INT → PF	0.145	0.022	7.272	0.000
SNS → INT → PF	0.054	0.013	4.109	0.000
PBC → INT → PF	0.096	0.015	6.304	0.000
CCI → INT → PF	0.015	0.017	0.913	0.361
CCG → INT → PF	0.031	0.018	1.809	0.070
Total	0.773	0.032	24.851	0.000
Use the feedback pathway.				
AAT → INT → UF	0.068	0.020	3.804	0.000
IAT → INT → UF	0.194	0.030	7.934	0.000
SNS → INT → UF	0.072	0.018	4.219	0.000
PBC → INT → UF	0.128	0.020	6.670	0.000
CCI → INT → UF	0.020	0.024	0.920	0.358
CCG → INT → UF	0.041	0.027	1.794	0.073
Total	0.793	0.039	22.204	0.000

Std. Est = Standardized parameter estimates; SE = Standard Error; SF pathway = Seek Feedback pathway; PF pathway = Process Feedback pathway; UF pathway = Use Feedback pathway.

Furthermore, the total effect coefficients on each action of students’ self-feedback were statistically significant, while the coefficients varied from 0.77 to 0.79, and the mean effect was 0.78. As indicated by the magnitude differences of indirect effects, the predictive effects were the strongest toward the Use Feedback pathway and the weakest toward the Process Feedback pathway.

## Discussion

This study adopted the extended TPB model to examine the predictive effects of students’ intentions and behaviors of self-feedback. The findings generally corroborated the two hypotheses: firstly, students’ self-feedback intentions could be predicted by their attitudes, subjective norms, and perceived behavioral control, but not by their perceived class climate concerning self-feedback; and secondly, students’ actual self-feedback behaviors could be determined by their intentions, perceived behavioral control, and partially by their perception of class climate.

### Scale re-formation

Notably, in the EFA study, the initial two sub-scales (CON and SEF) were suggested as one factor as PBC, which was consistent with the argument by the TPB inventor Ajzen (2020), “conceptually, there was no difference between perceived behavior control and self-efficacy” (Ajzen, 2020, p. 316). Both factors were used to predict individuals’ beliefs about performing certain behaviors, though, at the operational level, these two factors were measured differently. Self-efficacy was often measured as how participants foresee and overcome certain obstacles in completing certain behaviors. PBC was usually measured as the extent to which an individual had the capacity and belief to perform certain behaviors (Ajzen, 2012; Bandura, 1997). Meanwhile, the initial one-factor Class Climate was suggested to be divided into two factors (CCI and CCG), echoing Marsh et al.'s (2012) argument that it was crucial to distinguish the differences in perceived class climate at individual and group levels in educational studies. Specifically, the individual-level perception (CCI) measured the aggregations of each student; every individual in this cohort possessed his or her perception, motivation, and performance in specific learning environments. Since the referent here was the individual student, each student’s perceptions of class climate were inevitably different. Therefore, it was not interchangeable with other students in this cohort. On the contrary, group-level perception (CCG) measured a range of different variables characterized by a specific cohort in which each student was perceived. In other words, the referent was the entire class rather than individual students. It measured the general class climate perceived by the group from different perspectives. Therefore, this cohort’s perception was interchangeable. Furthermore, the items under CCI and CCG also supported the deconstruction of class climate. To exemplify it, the item of CCI2, *I feel good and comfortable in my class,* measured the class contextual factor experienced by individual students. In contrast, *in* the item of CCG3, *our teachers encourage us to ask questions when we do not understand* the climate experienced by the entire class.

### Predictors of self-feedback intention (RQ1)

This study’s three original TPB predictors significantly influenced students’ intention to self-feedback. However, the Class Climate factor (CCI and CCG) did not demonstrate any predictive significance to students’ intention of self-feedback. Furthermore, instrumental attitude (IAT, *β =* 0.36) and perceived behavior control (PBC, *β =* 0.24) were strong predictors. In contrast, the predictive power of subjective norms (SNS, *β = 0.14*) and affective attitude (AAT, *β =* 0.13) was weak, though still significant. The imperative role of attitude in determining behavioral intention is consistent with previous studies (Armitage and Conner, 2001; Yan and Sin, 2014; Yan and Cheng, 2015; Cooke et al., 2016; Yan et al., 2020). Instrumental attitude was the most effective predictor of students’ intention of self-feedback, which echoed previous studies investigating factors predicting students’ intention of self-assessment (Yan et al., 2020). It seemed that when students contemplated self-feedback, their intentions were notably influenced by the potential outcomes of their learning behaviors. Another crucial predictor of intention was perceived behavior control; this finding was consistent with previous studies (Armitage and Conner, 2001; Cooke et al., 2016; Yan et al., 2020). Self-efficacy appeared to be essential when students considered specific learning strategies, such as self-assessment and self-feedback. It was speculated that students’ decisions to engage in the self-feedback process are determined by comprehensive and practical considerations, such as the availability of relevant skills, requisite knowledge, sufficient time, and access to learning resources. This finding also implies that teachers shall prepare students with relevant knowledge and skills, and time and learning resources to gain more self-efficacy and become more inclined to engage in the self-feedback process.

In contrast, class climate was a non-significant factor in predicting students’ intention regarding self-feedback. This finding may be partially attributed to the broader scope of class climate compared with traditional TPB predictors. Class climate reflects students’ perceptions of their composite learning environment, encompassing the physical classroom setting and their psychological experiences, attitudes toward teachers and peers, and the quality of interpersonal interactions. Given this multidimensional nature, students may find it challenging to evaluate the learning environment concerning a specific behavioral outcome, such as self-feedback. Moreover, although classroom climate, regardless of individual or group level, was not identified as a significant predictor of intention to engage in self-feedback, this result underscores the need for further investigation. It is plausible that class climate produces its influence indirectly, for instance, through mediating factors such as student engagement, motivational processes, or metacognitive beliefs (Pintrich et al., 1993; Pintrich and Schragben, 2012). Future research could explore these pathways and potential interactions with teacher feedback practices or peer feedback culture to better understand how classroom context shapes students’ self-feedback processes. Furthermore, this finding suggested that neither gender nor grade level produced a significant moderating role in shaping students’ intention to engage in self-feedback; this was speculated that, in the practical feedback process, compared with the variations in gender and grade level, high school students might share more similarities in competitive learning experiences and intensive course content in mainland China (Wang, 2022; Chen et al., 2023; Miao and Ma, 2023).

### Predictors of self-feedback behavior (RQ2)

One innovative feature of this study was elaborating more specific and sequential behavioral elements (e.g., seeking, processing, and using feedback) of students’ self-feedback behaviors. Therefore, with a more specific and in-depth analysis of each action of self-feedback, it became possible for teachers and researchers to formulate a shared recognition and comprehension of students’ self-feedback; this would further shed light on the future motivation strategies for students’ self-feedback engagement in classroom instructional practices, as well as students’ capacity development of self-feedback. Therefore, it was imperative to explore the predictive effect of each factor on each action of self-feedback.

Both intention and PBC demonstrated significant predictive effects on students’ self-feedback; however, considering the predictive strength, intention is more influential than PBC in the present study, which was different from a previous study of self-assessment conducted by Yan et al. (2020), where they reported that PBC produced more substantial predictive effects on students’ self-assessment compared with intention. The possible reason is that self-assessment is primarily an assessment-oriented process, requiring students to evaluate their performance against established success criteria (Andrade, 2019; Panadero et al., 2016; Yan and Carless, 2021). Since this process is cognitively demanding, students must possess sufficient relevant knowledge, assessment skills, time, and access to specific resources. These requirements place a premium on perceived behavioral control, particularly self-efficacy, as students need confidence to generate accurate judgments to act on self-assessment tasks. In this context, it is reasonable that PBC emerged as more influential than intention in predicting self-assessment behaviors. In contrast, self-feedback is a learning-oriented process (Panadero et al., 2019, 2024). It involves students’ volitional beliefs to seek external information from teachers, peers, or the learning environment, compare and calibrate them with their personal learning experiences and performance, make learning inferences, and ultimately take initiatives to formulate their learning improvement strategies. While self-efficacy and controllability remain significant, this process relies more substantially on students’ willingness and motivational commitment to take the initiative to engage in the self-feedback process. As a result, intention becomes a stronger predictor of self-feedback than PBC. Moreover, this is consistent with the classical TPB assumption that intention is the most proximal determinant of behavior (Ajzen, 2020).

This finding aligned with prior research indicating that PBC consistently serves as a robust predictor of individuals’ behavior across various domains (Ajzen, 2020; Botetzagias et al., 2015; De Leeuw et al., 2015; Lizin et al., 2017; Ru et al., 2018; Yan et al., 2020). It was speculated that when students formulate self-feedback strategies, they consider internal elements, such as individual knowledge and skills relevant to the behavior, and external elements, such as the necessary time and financial support for the behavior’s completion. Additionally, this finding diverged from previous studies reporting that PBC holds greater predictive power than intention in students’ self-assessment (Yan et al., 2020) and math learning behavior (Wang et al., 2022). On one hand, it was speculated that students may be relatively less familiar with self-feedback strategies compared to self-assessment and math learning. Consequently, students were less experienced in evaluating the potential sources of control, including necessary knowledge and skills, as well as time and other resources required for executing self-feedback strategies. On the other hand, students require stronger volitional motivation to actively seek feedback from people and their surrounding environment, make sense of the received information, and take proactive agency to use this information to enhance their learning performance. Therefore, intention was more pivotal than perceived behavioral control in self-feedback.

A notable aspect of this study was the inclusion of class climate as an extra predictor in the conventional TPB model since class climate demonstrated a nuanced effect on self-feedback behavior. Specifically, at the individual level (CCI), it showed significant predictive power over students’ use of feedback but did not significantly predict their seeking and processing of feedback. Conversely, at the group level (CCG), it demonstrated significant predictive power in students’ seeking and sense-making of information but lacked predictive power in using feedback. As students actively seek and process information from their peers, teachers, friends, and other environmental sources, their interactions with the learning environment and individuals become crucial. Therefore, the overall class climate, encompassing a supportive classroom learning environment and sufficient learning resources, significantly impacted students’ seeking and processing of feedback behavior. This aligned with the socioecological perspective of the feedback process, which emphasizes that feedback was socially constructed and contextually situated (Boud and Molloy, 2013; Ajjawi and Boud, 2017; Chong, 2021). However, when students took proactive actions to revisit their learning goals and formulate learning growth plans, these were predominantly individual endeavors, which were likely influenced by their perception rather than their overall perception of the learning environment (Trickett and Moos, 1973; Marsh et al., 2012).

### Limitations and directions for future research

Though this study has contributed to novel exploration into the predictors of students’ intentions and behaviors related to self-feedback, further research is still necessary. First, the self-reported data of participants in this study might cause mono-method and response bias, including potential inaccuracies arising from memory limitations and social desirability effects. Future investigations may use direct and objective research methods, such as observations, to supplement or validate self-reported data to enhance the assessment accuracy of self-feedback behavior. Despite the inherent challenge in assessing attitudes, intentions, and internal processes related to self-feedback, forthcoming research might consider innovative methods, such as leveraging digital traces and eye-tracking techniques, to mitigate the shortcomings of self-report data. Second, this study examined the predictive effect of students’ intention and self-feedback behavior. However, the absence of data collection and analysis concerning academic proficiency hinders our comprehension of the predictive abilities of students’ self-feedback behavior on academic achievement. Therefore, future investigations should consider the collection of academic proficiency data as an outcome variable. Third, this study categorized students’ self-feedback behaviors into three sub-actions: seeking, processing, and using feedback. This fine-grained self-feedback behavior model allowed us to analyze and develop students’ intentions and capabilities for each action separately. However, students’ perceptions and intentions may influence each action’s unique role within the self-feedback process. As a result, this subdivision of behaviors might cause inconsistencies in the TPB model. Thus, building on the broad insights provided by this study, future research could refine its focus by examining each self-feedback action within the TPB framework. Fourth, the data for this study were collected from four high schools in Shenzhen, a prosperous coastal city in South China, which may limit the generalizability of the findings. To enhance the robustness and external validity of future research, it would be valuable to investigate the mechanisms of self-feedback behavior among students from more diverse geographical regions, across different educational levels, and within varied cultural contexts. Such efforts would allow for more comprehensive examinations and strengthen the applicability of the findings.

## Conclusion

This study makes both theoretical and practical contributions. Theoretically, the extended TPB model emerges as an appropriate framework for understanding self-feedback behavior, with intention playing a central role. Adopting the TPB model in self-feedback investigations enhances our understanding of how various individual factors influence students’ intentions and behaviors regarding self-feedback, establishing a basis for future research in this area. These findings can inform future pedagogical strategies employed by teachers to motivate students to engage in the self-feedback process and monitor students’ feedback behaviors. By implementing these strategies, students stand to benefit from the self-feedback process, thereby enhancing their learning efficacy and performance. School managers may also leverage these insights in their practical endeavors, such as designing teachers’ professional development courses focusing on adopting self-feedback as a classroom instructional strategy.

## Data Availability

The raw data supporting the conclusions of this article will be made available by the authors, without undue reservation.
