# Supplementary material for: Exploring factors influencing students’ self-feedback: insights from a structural equation modeling analysis using an extended theory of planned behavior framework
Source: Front Psychol. 2025 Oct 22;16:1683523. doi: 10.3389/fpsyg.2025.1683523 (PMC12586099; doi:10.3389/fpsyg.2025.1683523)
Supplement: Supplementary file 1 [file Supplementary_file_1.docx]

**Appendix 1**

*Factor Loadings for the final EFA with oblique rotation (direct oblimin) (Sample 1, N=656)*

| Rotated Factor Loadings | | | | | | | | | | | |
| --- | --- | --- | --- | --- | --- | --- | --- | --- | --- | --- | --- |
| Code | F 4 | F 9 | F 1 | F 7 | F 8 | F 10 | F 2 | F 5 | F 3 | F 6 | Communality |
| AAT1 |  |  | 0.49 |  |  |  |  |  |  |  | 0.66 |
| AAT2 |  |  | 0.72 |  |  |  |  |  |  |  | 0.85 |
| AAT3 |  |  | 0.90 |  |  |  |  |  |  |  | 0.97 |
| AAT4 |  |  | 0.43 |  |  |  |  |  |  |  | 0.73 |
| IAT1 |  |  |  |  |  |  | 0.46 |  |  |  | 0.59 |
| IAT4 |  |  |  |  |  |  | 0.52 |  |  |  | 0.84 |
| IAT5 |  |  |  |  |  |  | 0.54 |  |  |  | 0.71 |
| SNS1 |  |  |  |  |  |  |  |  | 0.59 |  | 0.64 |
| SNS2 |  |  |  |  |  |  |  |  | 0.70 |  | 0.65 |
| SNS3 |  |  |  |  |  |  |  |  | 0.52 |  | 0.46 |
| PBC1 | 0.55 |  |  |  |  |  |  |  |  |  | 0.55 |
| PBC2 | 0.83 |  |  |  |  |  |  |  |  |  | 0.76 |
| PBC3 | 0.68 |  |  |  |  |  |  |  |  |  | 0.75 |
| PBC4 | 0.83 |  |  |  |  |  |  |  |  |  | 0.81 |
| PBC6 | 0.51 |  |  |  |  |  |  |  |  |  | 0.59 |
| PBC7 | 0.47 |  |  |  |  |  |  |  |  |  | 0.58 |
| CCA1 |  |  |  |  |  |  |  | 0.75 |  |  | 0.63 |
| CCA2 |  |  |  |  |  |  |  | 0.76 |  |  | 0.78 |
| CCA3 |  |  |  |  |  |  |  | 0.70 |  |  | 0.69 |
| CCB1 |  |  |  |  |  |  |  |  |  | 0.63 | 0.63 |
| CCB2 |  |  |  |  |  |  |  |  |  | 0.60 | 0.56 |
| CCB3 |  |  |  |  |  |  |  |  |  | 0.67 | 0.65 |
| CCB4 |  |  |  |  |  |  |  |  |  | 0.43 | 0.56 |
| INT1 |  |  |  | 0.71 |  |  |  |  |  |  | 0.89 |
| INT2 |  |  |  | 0.53 |  |  |  |  |  |  | 0.80 |
| INT3 |  |  |  | 0.47 |  |  |  |  |  |  | 0.78 |
| INT5 |  |  |  | 0.51 |  |  |  |  |  |  | 0.76 |
| SF1 |  |  |  |  | 0.64 |  |  |  |  |  | 0.64 |
| SF2 |  |  |  |  | 0.57 |  |  |  |  |  | 0.51 |
| SF3 |  |  |  |  | 0.55 |  |  |  |  |  | 0.60 |
| SF4 |  |  |  |  | 0.62 |  |  |  |  |  | 0.70 |
| PF1 |  | 0.68 |  |  |  |  |  |  |  |  | 0.81 |
| PF2 |  | 0.68 |  |  |  |  |  |  |  |  | 0.75 |
| PF3 |  | 0.64 |  |  |  |  |  |  |  |  | 0.67 |
| UF2 |  |  |  |  |  | 0.66 |  |  |  |  | 0.70 |
| UF3 |  |  |  |  |  | 0.67 |  |  |  |  | 0.54 |
| UF4 |  |  |  |  |  | 0.47 |  |  |  |  | 0.59 |

Note. Factor loadings > 0.40 in boldface. Factor 1 = Affective Attitude; Factor 2 = Instrumental Attitude, Factor 3 = Subjective Norms, Factor 4 = Perceived Behavior Control, Factor 5 = Class Climate A, Factor 6 = Class Climate B; Factor 7= Intention of Self-feedback; Factor 8 = Seek Feedback; Factor 9 = Process Feedback; Factor 10 = Use Feedback.
