# Supplementary material for: Exploring factors influencing students’ self-feedback: insights from a structural equation modeling analysis using an extended theory of planned behavior framework
Source: Front Psychol. 2025 Oct 22;16:1683523. doi: 10.3389/fpsyg.2025.1683523 (PMC12586099; doi:10.3389/fpsyg.2025.1683523)
Supplement: Supplementary file 2 [file Supplementary_file_2.docx]

**Appendix 2**

| No | Code | Content |
| --- | --- | --- |
| 1 | AAT1 | I like self-feedback. |
| 2 | AAT2 | Self-feedback is an engaging experience for me. |
| 3 | AAT3 | I enjoy the process of self-feedback. |
| 4 | AAT4 | I feel confident practicing self-feedback. |
| 5 | IAT1 | Self-feedback gives an accurate appraisal of my performance. |
| 6 | IAT2 | *Self-feedback raises my interest in learning.* |
| 7 | IAT3 | *Self-feedback encourages me to be independent in my learning.* |
| 8 | IAT4 | Self-feedback helps me learn more efficiently. |
| 9 | IAT5 | Self-feedback raises my scores and grades. |
| 10 | *IAT6* | *Self-feedback helps me check my progress against achievement objectives.* |
| 11 | SNS1 | I believe my school principal wants all students to practice self-feedback. |
| 12 | SNS2 | I believe my teachers want me to do self-feedback. |
| 13 | SNS3 | Among my friends, we know self-feedback will help us learn. |
| 14 | SNS4 | *I believe my classmates take self-feedback seriously.* |
| 15 | PBC1 | I have the freedom whether or not to implement self-feedback. |
| 16 | PBC2 | The frequency of self-feedback is up to me. |
| 17 | PBC3 | I decide which method of self-feedback to use. |
| 18 | PBC4 | I am deciding when I should do self-feedback. |
| 19 | PBC5 | *I know the values of self-feedback.* |
| 20 | PBC6 | I know how to implement self-feedback. |
| 21 | PBC7 | I can find materials to conduct self-feedback. |
| 22 | *PBC8* | *I know how to approach others for feedback information.* |
| 23 | *PBC9* | *I can formulate appropriate methods to conduct self-feedback.* |
| 24 | *PBC10* | *I can tell whether my self-feedback practice is good or bad.* |
| 25 | CCI1 | In my class, students help decide good climate and discipline rules for the classroom. |
| 26 | CCI2 | I feel good and comfortable in my class. |
| 27 | CCI3 | In my class, we all get along with each other. |
| 28 | *CCI4* | *In my class, each student is accepted and valued for who he/she is.* |
| 29 | CCG1 | Our teachers tell us why we cannot do certain things. |
| 30 | CCG2 | Our teachers tell us that we can all learn, even if at different paces. |
| 31 | CCG3 | Our teachers encourage us to ask questions when we do not understand. |
| 32 | CCG4 | In my class, we try our best to do well. |
| 33 | INT1 | I willingly exercise self-feedback. |
| 34 | INT2 | Of my own accord, self-feedback is integrated into my learning. |
| 35 | INT3 | I am enthusiastic about making sure self-feedback is part of my studying. |
| 36 | *INT4* | *I am willing to design appropriate tasks for self-feedback.* |
| 37 | INT5 | I readily try to conduct self-feedback on myself. |
| 38 | *INT6* | *I plan to implement self-feedback through all courses in my learning.* |
| 39 | SF1 | I reflect on the quality of my own work and use my reflection as a source of information to improve my work. |
| 40 | SF2 | I seek out examples of good work to improve my work. |
| 41 | SF3 | I ask for comments about specific aspects of my work from others. |
| 42 | SF4 | When other people provide me with input about my work I listen or read thoughtfully. |
| 43 | PF1 | I carefully consider comments about my work before deciding if I will use them or not. |
| 44 | PF2 | When receiving conflicting information about my work from different sources, I make a judgement about what I will use. |
| 45 | PF3 | When deciding what to do with comments, I consider the credibility of their sources. |
| 46 | *UF1* | *I can formulate my learning improvement plan after explicit inferences.* |
| 47 | UF2 | I would spend more time working on my advantageous areas. |
| 48 | UF3 | I would spend more time working on my weak areas. |
| 49 | UF4 | When receiving comments I plan how to use them to improve my future work, not just the immediate task. |

*Note.* The items in underlined italics were removed from the final scale
