# Supplementary material for: Exploring factors influencing students’ self-feedback: insights from a structural equation modeling analysis using an extended theory of planned behavior framework
Source: Front Psychol. 2025 Oct 22;16:1683523. doi: 10.3389/fpsyg.2025.1683523 (PMC12586099; doi:10.3389/fpsyg.2025.1683523)
Supplement: Supplementary file 3 [file Supplementary_file_3.docx]

**Appendix 3**

*Factor loadings report of each scale.*

| Factor | Std.Est | SE | z | P |
| --- | --- | --- | --- | --- |
| AAT =~ |  |  |  |  |
| AAT1 | 0.817 |  |  |  |
| AAT2 | 0.935 | 0.027 | 42.816 | 0.000 |
| AAT3 | 0.942 | 0.028 | 43.335 | 0.000 |
| AAT4 | 0.802 | 0.031 | 33.702 | 0.000 |
| IAT =~ |  |  |  |  |
| IAT1 | 0.761 |  |  |  |
| IAT4 | 0.919 | 0.034 | 36.113 | 0.000 |
| IAT5 | 0.866 | 0.035 | 33.569 | 0.000 |
| SNS =~ |  |  |  |  |
| SNS1 | 0.771 |  |  |  |
| SNS2 | 0.761 | 0.033 | 27.219 | 0.000 |
| SNS3 | 0.817 | 0.036 | 29.338 | 0.000 |
| PBC =~ |  |  |  |  |
| PBC1 | 0.826 |  |  |  |
| PBC2 | 0.868 | 0.028 | 38.642 | 0.000 |
| PBC3 | 0.898 | 0.026 | 40.908 | 0.000 |
| PBC4 | 0.879 | 0.026 | 39.463 | 0.000 |
| PBC6 | 0.819 | 0.028 | 35.247 | 0.000 |
| PBC7 | 0.754 | 0.030 | 31.240 | 0.000 |
| CCI =~ |  |  |  |  |
| CCI1 | 0.763 |  |  |  |
| CCI2 | 0.873 | 0.034 | 31.984 | 0.000 |
| CCI3 | 0.857 | 0.031 | 31.449 | 0.000 |
| CCG =~ |  |  |  |  |
| CCG1 | 0.827 |  |  |  |
| CCG2 | 0.811 | 0.033 | 32.435 | 0.000 |
| CCG3 | 0.755 | 0.031 | 29.536 | 0.000 |
| CCG4 | 0.728 | 0.033 | 28.182 | 0.000 |
| INT =~ |  |  |  |  |
| INT1 | 0.889 |  |  |  |
| INT2 | 0.896 | 0.019 | 48.016 | 0.000 |
| INT3 | 0.907 | 0.019 | 49.336 | 0.000 |
| INT5 | 0.837 | 0.023 | 41.420 | 0.000 |
| SF =~ |  |  |  |  |
| SF1 | 0.782 |  |  |  |
| SF2 | 0.802 | 0.033 | 31.021 | 0.000 |
| SF3 | 0.722 | 0.039 | 27.258 | 0.000 |
| SF4 | 0.718 | 0.033 | 27.077 | 0.000 |
| PF =~ |  |  |  |  |
| PF1 | 0.850 |  |  |  |
| PF2 | 0.831 | 0.027 | 37.441 | 0.000 |
| PF3 | 0.857 | 0.025 | 39.512 | 0.000 |
| UF =~ |  |  |  |  |
| UF2 | 0.789 |  |  |  |
| UF3 | 0.696 | 0.034 | 24.999 | 0.000 |
| UF4 | 0.791 | 0.034 | 28.898 | 0.000 |

*Note*. AAT: Affective attitude; IAT: Instrumental attitude; SNS: Subjective norm; PBC: Perceived Behavior Control; CCI: Class Climate at Individual Level; CCG: Class Climate at Group Level; INT: Intention; SF: Seek Feedback; PF: Process Feedback; UF: Use Feedback.
